# Midlife Vascular and Lifestyle Determinants of Late-Life Cognitive Decline and Dementia: A Life-Course Prevention Framework with a Gulf (GCC) Perspective

**DOI:** 10.3390/life16081289

**Published:** 2026-08-05

**Authors:** Najeeb Qadi, Amaal Aldakheel, Eslam Shosha

**Affiliations:** 1 Neuroscience Centre of Excellence, King Faisal Specialist Hospital and Research Centre, P.O. Box 3354, Riyadh 11211, Saudi Arabia; adakheel@kfshrc.edu.sa (A.A.); eshosha@kfshrc.edu.sa (E.S.); 2 College of Medicine, Alfaisal University, P.O. Box 50927, Riyadh 11533, Saudi Arabia; 3 Division of Neurology, Department of Medicine, McMaster University, Hamilton, ON L8N 3Z5, Canada

**Keywords:** dementia, cognitive decline, midlife, vascular risk, lifestyle, prevention, Alzheimer’s disease, cognitive reserve, brain health, Gulf Cooperation Council

## Abstract

Dementia is a growing global health challenge, yet many determinants of late-life cognitive decline emerge decades before symptoms appear. Midlife is a practical window for prevention because hypertension, diabetes, obesity, dyslipidemia, smoking, physical inactivity, unhealthy diet, sleep disturbance, and social isolation can be identified and modified before substantial brain injury becomes apparent. This narrative review synthesizes evidence linking midlife vascular and lifestyle exposures to late-life cognitive impairment and dementia. The most consistent data support a life-course model in which cumulative vascular, metabolic, inflammatory, and behavioral risks interact with neurodegenerative pathology and cognitive reserve. Vascular and metabolic factors act largely through small-vessel disease, endothelial dysfunction, and inflammation, whereas physical activity, healthy diet, sleep, and social engagement may strengthen resilience. Although observational evidence is vulnerable to confounding and single-risk trials may underestimate cumulative benefit, multidomain prevention remains biologically plausible and clinically actionable, as recent trials reaffirm. These priorities are especially salient in the rapidly transitioning Gulf Cooperation Council (GCC) countries, where midlife cardiometabolic risk is high and local evidence is limited. Dementia prevention should be embedded in routine midlife care, with vascular risk management and sustained lifestyle support treated as core elements of lifelong brain health.

## 1. Introduction

Dementia is a major cause of disability, dependency, and death among older adults. The World Health Organization estimated that 57 million people were living with dementia in 2021, with nearly 10 million new cases each year and a disproportionate burden in low- and middle-income countries [1]. Because disease-modifying treatments remain limited in availability, indications, and population-level impact, prevention has become a central priority for clinical medicine and public health.

The 2024 Lancet Commission on dementia prevention, intervention, and care strengthened this preventive perspective by estimating that approximately 45% of dementia cases worldwide might be preventable or delayable by addressing 14 modifiable risk factors across the life course [2]. Several of these risks become established in midlife, including hypertension, obesity, high low-density lipoprotein cholesterol, excessive alcohol use, traumatic brain injury, and other cardiometabolic and behavioral exposures [2]. This timing matters because midlife is early enough to permit sustained risk reduction yet late enough for vascular and metabolic abnormalities to be measurable in routine practice.

Dementia should therefore not be viewed solely as a disorder of old age. Alzheimer’s disease and related dementias may have long preclinical phases during which amyloid, tau, synaptic dysfunction, vascular injury, inflammation, and network-level changes accumulate before symptoms become clinically apparent [3]. Midlife vascular and lifestyle determinants may modify this trajectory by accelerating brain injury, lowering cognitive reserve, or increasing resilience. This review examines clinically actionable midlife determinants of late-life cognitive decline and dementia, with an emphasis on vascular and lifestyle risks that can be addressed in routine adult care.

The case for prevention is especially pressing in regions undergoing rapid epidemiological transition. While several high-income countries have reported stable or declining age-specific dementia rates, the steepest future increases are projected for North Africa and the Middle East, where the number of people living with dementia is expected to rise by roughly 367% between 2019 and 2050, the largest increase in any world region, driven by population aging and rising obesity, diabetes, and smoking [4]. Because the exposures driving that projection are precisely the ones that are measurable and modifiable in midlife, the life-course prevention argument may carry particular weight in the Gulf Cooperation Council (GCC) countries (Saudi Arabia, the United Arab Emirates, Qatar, Kuwait, Bahrain, and Oman) and in comparable settings. The regional epidemiology, genetic background, and delivery implications are examined in Section 7; the intervening sections set out the general life-course framework on which that discussion builds.

## 2. Scope and Approach

This article is a narrative review, not a systematic review or meta-analysis. It synthesizes landmark cohort studies, randomized trials, systematic reviews, and major public health reports relevant to midlife vascular and lifestyle determinants of later cognitive outcomes. No new human or animal data were generated or analyzed. The review prioritizes exposures common in clinical practice and potentially modifiable: hypertension, diabetes and insulin resistance, obesity, dyslipidemia, smoking, physical inactivity, diet, sleep, cognitive engagement, and social connection.

The term midlife is used pragmatically to refer to the period of adulthood before older age, often between 40 and 65 years, acknowledging that definitions vary across cohorts. The term dementia is used clinically and etiologically to include Alzheimer’s disease, vascular dementia, mixed dementia, and other late-life cognitive disorders in which vascular and lifestyle determinants may influence risk or expression. Because many exposures cluster, the review emphasizes cumulative and multidomain risk rather than deterministic claims about any single factor.

Consistent with a narrative review, the literature was selected purposively rather than through a predefined systematic search. To make that process transparent, we proceeded in three steps. First, for each exposure we anchored the evidence on the major consensus reports and landmark population-based cohorts that define the field, together with any randomized trials reporting cognitive or dementia endpoints. Second, we searched PubMed and Google Scholar for records published through June 2026, combining terms for each exposure (hypertension, diabetes, obesity, dyslipidemia, smoking, physical activity, diet, sleep, cognitive engagement, and social connection) with terms for cognitive decline, dementia, and Alzheimer’s disease, and screened titles and abstracts for relevance; regionally relevant GCC studies were identified by the same strategy. Third, we hand-searched the reference lists of retrieved reviews and trials. Where several studies addressed the same question, we prioritized the most recent systematic review or meta-analysis, the largest or longest-followed cohort, and studies reporting clinically interpretable endpoints. Studies were not formally scored, no protocol was registered, and no quantitative synthesis was undertaken; the intent was an integrative, clinically oriented synthesis rather than a pooled estimate.

Because the evidence base is uneven across exposures, we distinguish throughout between findings supported by intervention studies and those resting mainly on observational data. Among the vascular exposures, blood-pressure lowering has the strongest interventional evidence, and multidomain lifestyle programs have been tested as packages rather than as separable components; most remaining associations, including those for adiposity, diet, sleep, smoking, and social engagement, derive predominantly from cohort studies and are therefore vulnerable to residual confounding and reverse causation. Mendelian randomization contributes an intermediate class of evidence for selected exposures by approximating lifelong differences in a risk factor. This hierarchy is stated explicitly for each domain in Table 1 and is carried through to the conclusions, where recommendations resting on trial evidence are separated from those that remain biologically plausible but unproven.

## 3. Dementia as a Life-Course Disorder

A life-course model is useful because dementia risk reflects both accumulated injury and accumulated reserve. Early-life education, midlife vascular and metabolic health, and late-life sensory, social, and functional factors may interact over decades [2,34,35]. From this perspective, late-life cognitive decline may represent the clinical endpoint of long-standing biological vulnerability rather than the sudden onset of disease in old age.

Midlife is a particularly strategic period. Blood pressure, glycemic status, body weight, lipid levels, smoking exposure, sleep quality, and exercise habits are routinely measurable and often modifiable. Importantly, vascular and metabolic injury can begin before overt cognitive symptoms appear, and imaging studies suggest that midlife vascular risk factors may be linked to later brain changes, including amyloid deposition and structural injury [36,37]. Therefore, prevention efforts that wait until dementia symptoms appear may miss the window when vascular and lifestyle modifications can meaningfully alter long-term trajectories.

This framework also explains why interventions in older adults can yield heterogeneous results. Once neuropathology is established, risk-factor control remains important for general health and may slow vascular injury, but it may be less effective at reversing existing neurodegeneration.

## 4. Midlife Vascular Determinants

### 4.1. Hypertension and Cerebral Small-Vessel Disease

Hypertension is among the most consistent midlife vascular predictors of later cognitive impairment and dementia. Large observational cohorts have linked elevated midlife blood pressure to an increased risk of subsequent dementia, and risk appears to rise when hypertension co-occurs with other cardiometabolic exposures [5,6]. Mechanistically, chronic hypertension promotes arteriolar sclerosis, endothelial dysfunction, impaired autoregulation, disruption of the blood–brain barrier, white matter injury, lacunes, microbleeds, and reduced cerebrovascular reactivity. These processes can directly impair attention, processing speed, executive function, and gait and may also lower the threshold for the clinical expression of Alzheimer’s pathology.

The randomized SPRINT MIND trial did not show a statistically significant reduction in probable dementia, its primary cognitive outcome, but intensive systolic blood pressure control reduced mild cognitive impairment and the combined outcome of mild cognitive impairment or probable dementia [9]. This pattern is clinically important: it supports the concept that vascular risk management can influence cognitive trajectories and reminds clinicians that dementia is multifactorial and may require longer follow-up or multidomain interventions. In midlife practice, blood pressure control should be framed not only as cardiovascular protection but also as preservation of brain health.

Recent large-scale evidence continues to refine this picture. A 2025 analysis of 1.3 million UK women found that midlife hypertension was associated with increased dementia risk, though the strength of this association varied appreciably across cohorts and dementia subtypes, underscoring that the association is consistent but heterogeneous rather than uniform [7]. A 2024 systematic review and meta-analysis similarly concluded that midlife hypertension affects some cognitive domains, particularly memory, executive function, and global cognition, more consistently than others, suggesting that blood-pressure-related injury may not impair all aspects of cognition equally [8]. These nuances do not weaken the case for blood pressure control; rather, they argue for more precise risk communication that distinguishes domain-specific effects from a uniform reduction in dementia risk.

### 4.2. Diabetes Mellitus and Insulin Resistance

Diabetes mellitus and insulin resistance are linked to an increased risk of cognitive decline and dementia through overlapping metabolic and vascular pathways [5,10]. Hyperglycemia contributes to oxidative stress, advanced glycation end-products, endothelial dysfunction, inflammation, and microvascular injury. Insulin resistance may also affect neuronal metabolism, synaptic signaling, and amyloid- or tau-related pathways, although the extent to which Alzheimer’s disease should be conceptualized as a metabolic disorder remains debated.

Diabetes rarely occurs in isolation. It commonly coexists with obesity, hypertension, dyslipidemia, chronic kidney disease, sleep-disordered breathing, and physical inactivity. These clustered risks may exacerbate cerebral small-vessel disease and systemic inflammation. Therefore, brain-health counseling for patients with diabetes should extend beyond glucose control and include integrated management of blood pressure, lipids, weight, exercise, sleep, and smoking status.

Recent evidence has refined this picture. Glycemic variability and longer diabetes duration are associated with higher dementia risk independent of mean glucose control, implicating fluctuations themselves, not only chronic hyperglycemia [11]. Current reviews confirm that diabetes-related cognitive impairment operates through overlapping pathways of oxidative stress, neuroinflammation, and brain insulin resistance, with lifestyle interventions the most consistently supported mitigation [12,38]. Insulin resistance assessed independently of overt diabetes has been linked to accelerated brain atrophy and cognitive decline, suggesting the relevant exposure may begin before diabetes is diagnosed [13,39].

### 4.3. Obesity, Adiposity, and Metabolic Syndrome

Midlife obesity, particularly central adiposity, has been linked to an increased risk of late-life dementia in several epidemiological studies [6,14]. Adipose tissue is metabolically active and can promote insulin resistance, chronic low-grade inflammation, altered adipokine signaling, oxidative stress, and endothelial dysfunction. These processes may contribute to both vascular brain injury and neurodegenerative vulnerability.

The timing of body weight measurement is important. Midlife obesity appears more consistently harmful than late-life body mass index, partly because unintentional weight loss can occur during the preclinical phase of dementia and may obscure causal direction. Clinically, weight management should be positioned as part of cardiometabolic and cognitive prevention rather than as a purely cosmetic or metabolic goal. The strongest message is not that weight alone determines dementia risk, but that adiposity contributes to a broader network of modifiable exposures.

Updated reviews support this relationship while clarifying its boundaries. A 2025 meta-analysis of nearly three million older adults found the association strongest for central rather than general adiposity [15]. Neuroimaging studies show that midlife visceral and subcutaneous abdominal fat predict later brain volume loss, and adverse body composition patterns track with neurodegenerative risk in UK Biobank data [40,41]. Body weight variability, not absolute weight alone, may also carry independent prognostic value beyond a single BMI measurement, a pattern confirmed in a 2025 meta-analysis of more than four million participants [16,42].

### 4.4. Dyslipidemia and Low-Density Lipoprotein Cholesterol

The relationship between lipids and dementia is complex and varies by lipid fraction, age at measurement, vascular comorbidity, and outcome definition. Nevertheless, high low-density lipoprotein cholesterol in midlife was added as a modifiable dementia risk factor in the 2024 Lancet Commission, reinforcing the importance of lipid control as part of a life-course prevention strategy [2]. Dyslipidemia may contribute to atherosclerosis, impaired cerebral perfusion, endothelial dysfunction, and vascular inflammation. It may also interact with obesity, diabetes, and hypertension to increase cumulative brain injury.

Current evidence does not justify presenting lipid-lowering as a guaranteed dementia prevention treatment. Rather, dyslipidemia should be managed according to cardiovascular-risk standards, with the additional message that vascular protection is relevant to future cognition. This balanced framing avoids overstatement and strengthens patients’ motivation to sustain risk-factor control.

The apparent discrepancy between a risk factor endorsed by the Lancet Commission [2] and trial evidence that does not support lipid lowering for cognition [17] deserves explanation rather than simple acknowledgment, because the two bodies of evidence address different exposures. The observational and commission-level evidence concerns cumulative LDL-C exposure beginning in midlife and extending across decades, whereas the randomized trials enrolled participants at a mean age of roughly 65 years and followed them for a median of about three years. A neutral trial result therefore tests late, short-duration lipid lowering rather than the sustained midlife exposure to which the risk-factor designation refers. Genetic evidence supports this reading: a 2025 Mendelian randomization analysis of more than one million individuals found that genetically proxied lifelong lowering of non-HDL cholesterol, acting through the targets of statins, ezetimibe, and CETP inhibitors, was associated with a reduced risk of dementia [18]. Because genetic variants approximate lifelong exposure whereas trials capture only a few years of treatment late in life, the discordance is more plausibly explained by the timing and duration of the intervention window than by an absence of biological relevance for midlife LDL-C. The practical implication for patients is unchanged: lipid lowering should not be offered as a proven dementia prevention therapy. The rationale for treating midlife dyslipidemia early is nonetheless strengthened, rather than weakened, by the neutral late-life trials.

### 4.5. Smoking and Cumulative Vascular Injury

Smoking is a major modifiable contributor to vascular disease, oxidative stress, endothelial injury, thrombosis, chronic hypoxia, and systemic inflammation. These mechanisms provide a plausible pathway from tobacco exposure to later cognitive impairment, and epidemiological syntheses support an association between smoking and dementia risk [5,19]. Smoking also clusters with other health behaviors, leading to cumulative exposure to poor diet, inactivity, sleep disturbance, and lower engagement in preventive care in some populations.

Smoking cessation is therefore one of the highest-yield midlife interventions. Its proven benefits for cardiovascular, respiratory, cancer, and mortality outcomes are sufficient justification, and potential cognitive benefits further strengthen the case. In clinical practice, smoking status should be treated as a brain-health vital sign and addressed through repeated counseling, pharmacotherapy when appropriate, and system-level cessation support.

Recent large-cohort data have clarified the benefits of smoking cessation for cognitive outcomes. A 2026 analysis of more than 32,000 adults followed for up to 25 years found that smoking cessation was associated with a steadily declining dementia risk that approached that of never-smokers within five to seven years, although this benefit was attenuated among individuals who gained substantial weight after quitting [20]. A complementary multinational analysis across 12 countries similarly found that more favorable cognitive trajectories were observed after cessation, even when quitting occurred in mid- to late life [21]. Population-level modeling underscores the magnitude of this opportunity: smoking remains among the dementia risk factors with the largest estimated population-attributable fraction globally, alongside hypertension, obesity, and physical inactivity [43].

## 5. Midlife Lifestyle Determinants

### 5.1. Physical Activity and Fitness

Regular physical activity is among the most actionable lifestyle factors for long-term brain health. Observational evidence from midlife cohorts and meta-analyses links leisure-time physical activity to a lower risk of later cognitive decline, dementia, and Alzheimer’s disease [22,23]. Exercise may reduce dementia risk indirectly by lowering blood pressure, improving insulin sensitivity, promoting weight control, improving lipid profiles, reducing systemic inflammation, and improving sleep and mood. It may also exert more direct neurobiological effects through increased cerebral perfusion, neurotrophic signaling, synaptic plasticity, and hippocampal function.

The clinical challenge is implementation, not conceptual acceptance. Brief advice alone is often insufficient. Patients are more likely to sustain activity when recommendations are specific, realistic, and integrated with comorbidity management. Walking programs, resistance training, balance exercises, and supervised rehabilitation can be tailored to age, frailty, orthopedic limitations, and cultural context. For midlife patients, the goal should be decades of sustainable activity rather than short-term, intensive programs that cannot be maintained.

A comprehensive meta-analysis incorporating quality assessment of cohort and case–control evidence confirmed that physical activity is associated with a lower incidence of dementia and Alzheimer’s disease. The strength of these associations increased with longer follow-up, addressing earlier concerns that shorter studies might reflect reverse causation, in which preclinical decline reduces activity levels rather than activity protecting against decline [24].

### 5.2. Dietary Patterns and Cardiometabolic Health

Diet affects cognitive risk primarily through cardiometabolic and inflammatory pathways. Dietary patterns rich in vegetables, fruits, legumes, whole grains, nuts, fish, and unsaturated fats are associated with better vascular and metabolic profiles and have been linked to cognitive outcomes in observational studies [44,45]. The Mediterranean and Mediterranean-DASH Intervention for Neurodegenerative Delay (MIND) diets are often discussed because they align cardiovascular and cognitive prevention goals.

Diet should not be presented as a stand-alone dementia cure. Evidence is stronger for overall dietary patterns and cardiometabolic health than for isolated supplements or single nutrients. Clinically, dietary counseling is most credible when integrated into broader management of hypertension, diabetes, obesity, dyslipidemia, and physical inactivity. This approach also reduces the risk of overclaiming causality from observational nutrition studies.

More recent cohort and meta-analytic evidence has refined understanding of dietary patterns and cognitive outcomes. A 2023 meta-analysis of prospective cohorts found that MIND diet adherence was associated with better-preserved global cognition and slower decline, with effects evident across multiple study populations [46]. Subsequent work in the REGARDS cohort linked higher adherence to a MIND-style diet with a reduced risk of incident cognitive impairment. Analyses in racially diverse populations found that associations between the MIND diet and cognitive decline held in both Black and White older adults, though effect sizes varied by population, highlighting the importance of validating dietary recommendations across diverse groups rather than assuming uniform benefit [47,48]. A 2024 review in Nature Reviews Neurology further emphasized that dietary pattern, rather than any single nutrient or supplement, remains the most defensible target for brain-healthy nutritional counseling [49].

### 5.3. Sleep and Sleep-Disordered Breathing

Sleep has become an increasingly important component of brain health and prevention. Longitudinal evidence links short sleep duration in midlife to later dementia incidence [27]. Mechanistically, sleep disturbance may contribute to impaired glymphatic clearance, inflammation, vascular stress, insulin resistance, mood symptoms, and daytime cognitive inefficiency. One proposed mechanism is glymphatic clearance: experimental work, conducted largely in rodents, has shown that sleep can facilitate the removal of metabolites from the brain [28]. We treat this as a hypothesis rather than an established mechanism, because its extension to the human brain remains unsettled; the supporting evidence and its limitations are examined in Section 9. Importantly, the epidemiological association between disturbed sleep and dementia does not depend on the glymphatic account being correct, and the clinical case for identifying and treating sleep disorders rests on established benefits rather than on this mechanism.

Sleep-disordered breathing warrants particular attention because it is common, underdiagnosed, and linked to intermittent hypoxia, sympathetic activation, hypertension, atrial fibrillation, metabolic dysfunction, and daytime cognitive symptoms. Although definitive dementia prevention trials of sleep interventions remain limited, identifying and treating clinically significant sleep disorders is justified by established benefits in quality of life, safety, cardiometabolic health, and daytime function.

Recent meta-analytic syntheses have strengthened confidence in the sleep-dementia relationship and clarified its structure. A 2025 meta-analysis of sleep disorders found that obstructive sleep apnea, insomnia, and other sleep disturbances were each independently associated with increased risk of all-cause dementia and Alzheimer’s disease [29]. Large prospective cohort data from Japan have similarly linked both short sleep duration and adverse changes in sleep duration over time to elevated dementia incidence. An updated systematic review through 2025 confirmed that multiple distinct sleep parameters, including fragmentation and prolonged sleep latency, carry independent risk signals rather than reflecting a single underlying construct [30,50]. These findings support sleep assessment as a multidimensional component of brain-health screening rather than a single yes/no inquiry about sleep duration.

### 5.4. Cognitive Engagement, Social Connection, and Reserve

Cognitive reserve helps explain why individuals with similar neuropathological burdens may differ in clinical expression. Education, occupational complexity, lifelong learning, multilingualism, cognitively stimulating activities, and social participation may increase resilience or compensate for brain injury [31]. Social relationships may also protect cognition by reducing loneliness and depression, sustaining complex communication, supporting adherence to medical care, and promoting meaningful activity [32].

These factors are not purely individual choices. Educational opportunity, retirement patterns, family structure, urban design, disability access, and socioeconomic context all shape cognitive and social engagement. Therefore, prevention strategies should integrate individual counseling with community and policy approaches that promote social participation, lifelong learning, and access to meaningful activities throughout adulthood.

The evidence for the importance of social connection has grown considerably. The largest meta-analysis to date (>600,000 individuals, 21 cohorts) found that loneliness was associated with increased risk of all-cause dementia, Alzheimer’s disease, and vascular dementia. This association persisted after adjusting for depression and isolation, indicating that loneliness carries risk distinct from having few contacts [33]. Cohort data from thirteen international studies linked broader social connectedness to reduced dementia risk, and social isolation independently predicted nine-year risk among US community-dwelling adults [51,52]. These findings support routine screening for both loneliness and objective isolation as distinct, modifiable targets.

The major modifiable vascular and lifestyle determinants discussed in Section 4 and Section 5 are summarized in Table 1, together with an explicit indication of the class of evidence supporting each domain.

Table 2 highlights selected landmark studies that inform this prevention framework.

## 6. Integrated Mechanisms Linking Midlife Exposures to Late-Life Outcomes

Midlife vascular and lifestyle determinants converge through several interacting mechanisms. Cerebral small-vessel disease is central: hypertension, diabetes, smoking, dyslipidemia, and obesity can impair endothelial function, autoregulation, and perfusion, leading to white-matter hyperintensities, lacunes, microinfarcts, microbleeds, and network disconnection. These injuries may independently cause cognitive impairment and may also reduce the amount of Alzheimer pathology needed to produce clinical symptoms.

Metabolic and inflammatory pathways provide a second mechanism. Insulin resistance, adiposity, smoking, poor diet, sleep disruption, and inactivity can increase oxidative stress and chronic, low-grade inflammation. These processes may exacerbate vascular injury, impair mitochondrial function, disrupt synaptic signaling, and influence amyloid- or tau-related pathways. The result is not a single disease mechanism but a cumulative biological environment that renders the brain more vulnerable to aging and neurodegeneration.

**Figure 1:**
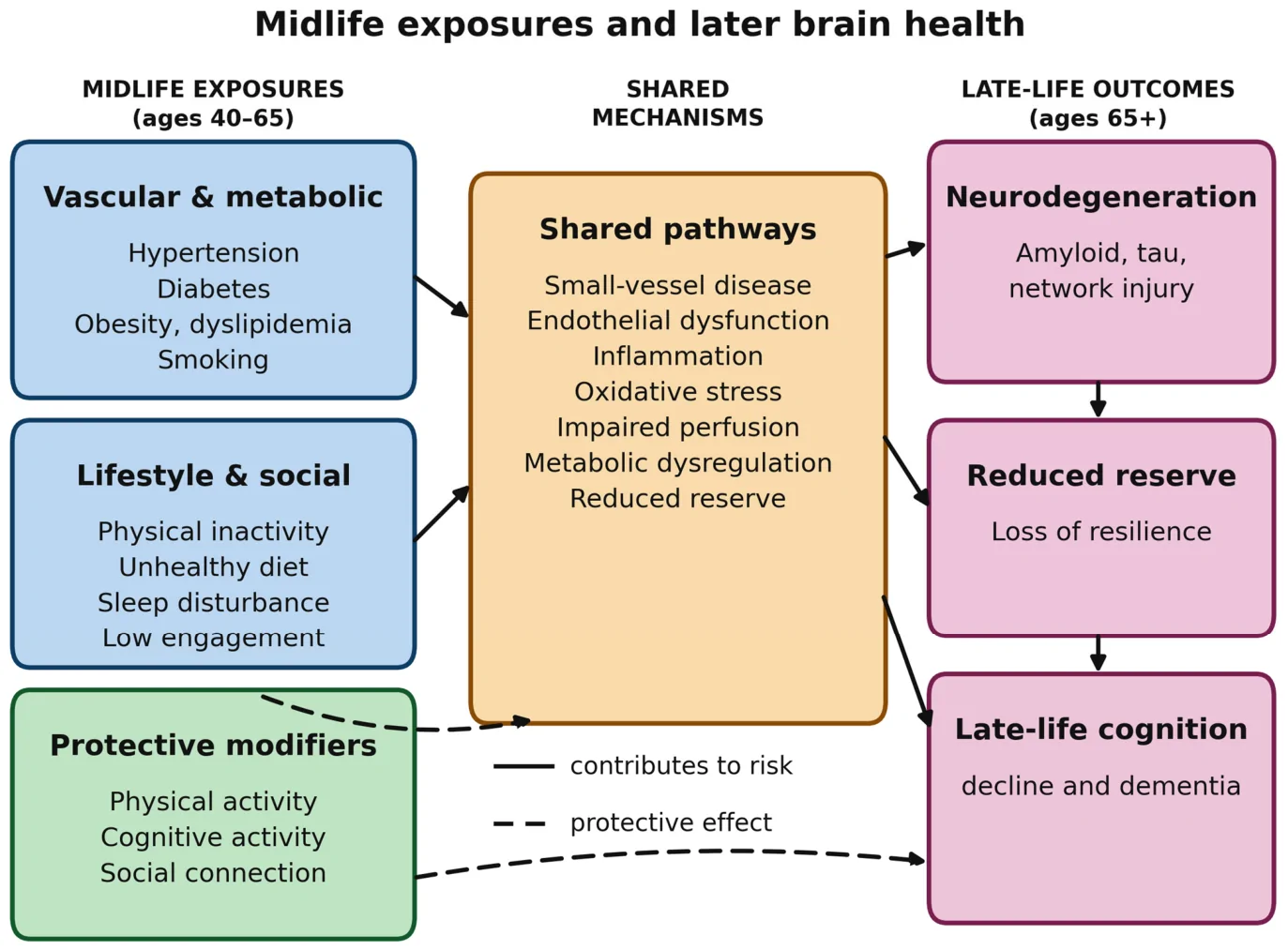
Conceptual framework linking midlife vascular and lifestyle exposures to late-life cognitive decline and dementia. The diagram summarizes plausible pathways rather than causal proof for each individual exposure. Midlife risks may act through vascular, inflammatory, metabolic, and reserve-related mechanisms, with cumulative and interacting effects over time.

Reserve and resilience constitute the third mechanism. Physical activity, cognitive stimulation, education, social connection, and healthy sleep may not eliminate pathology, but they can increase the capacity to tolerate or compensate for injury. Prevention is therefore best understood as a dual strategy: reduce cumulative injury while strengthening reserve. Figure 1 summarizes these interacting pathways.

## 7. Regional Relevance: Midlife Prevention in the Gulf (GCC) Countries

### 7.1. The Regional Burden of Risk and Disease

The life-course framework has particular implications for regions undergoing rapid epidemiological transition. Much of the cohort and trial evidence summarized above comes from North American and European populations, yet the demographic and cardiometabolic context of the Gulf Cooperation Council countries differs in ways that directly affect dementia risk. The region combines a young but rapidly aging population with an unusually high prevalence of midlife obesity, type 2 diabetes, physical inactivity, and tobacco use, so the very exposures linked to later cognitive decline are widespread decades before old age [4,53].

Local epidemiological data reinforce this picture. A retrospective analysis of dementia prevalence in Saudi Arabia’s National Guard Health System from 2015 to 2023 documented the rising burden of diagnosed dementia consistent with regional demographic aging, while highlighting the fragmentation and limited scope of routine surveillance data across Gulf health systems [54]. A synthesis of dementia prevalence and economic burden across Arab countries similarly concluded that the region faces a substantial and likely underestimated burden, compounded by a scarcity of large, methodologically rigorous, population-based studies compared with North American and European settings [55]. Health-economic modeling projects that Saudi Arabia’s non-communicable disease burden, including dementia, will grow substantially between 2020 and 2030 as the population over 65 nearly doubles relative to the working-age population, with dementia among the costliest conditions to manage at the national level [56]. A broader synthesis covering the wider Middle East reports the same pattern of rising burden, significant caregiving and economic costs, and persistent gaps in standardized data [57]. The case for midlife prevention in the Gulf therefore rests not only on extrapolation from global trends but also on emerging local evidence pointing in the same direction.

The distribution of the specific midlife exposures reviewed above is equally striking. Diabetes prevalence is among the highest worldwide, with national surveys reporting adult diabetes at roughly 18% in Qatar and comparably high levels across the other GCC states [56,58]. Adult obesity affects roughly one-third of the population in Qatar, and cardiometabolic risk clustering (central obesity together with dyslipidemia and dysglycemia) is already evident among young adults in the United Arab Emirates, well before midlife [58,59]. Regional data indicate that a large share of adults are insufficiently active, driven in part by climate and the built environment [60]. Tobacco use, including regionally specific forms such as waterpipe (shisha) smoking, remains common, and secondhand smoke exposure is widespread among adolescents across the GCC states [61]. Taken together, these exposures place a large share of the midlife population on a trajectory of cumulative cerebro-metabolic risk decades before old age, which is precisely the pattern the life-course model predicts will surface as dementia a generation later.

Saudi Arabia illustrates both the challenge and the opportunity. As longevity rises and the share of older adults grows, the prevalence of cognitive impairment is already substantial: the Riyadh community study noted above reported impairment in a large proportion of older primary-care patients, clustering with hypertension and cardiovascular disease [62]. At the same time, dementia research from the region remains limited, with few large population-based studies and little longitudinal or biomarker data [53]. This combination of a high modifiable-risk burden, a compressed demographic transition, and sparse local evidence means that midlife prevention could avert a disproportionate share of future cases, while also implying that prevention models cannot be imported unchanged.

### 7.2. Genetic Background and Consanguinity

The regional argument is usually framed in environmental terms, but the population genetics of the Gulf may also shape how midlife vascular risk translates into cognitive outcomes, and this dimension has received less attention than it deserves. Two features are relevant. First, APOE ε4, the principal common susceptibility allele for late-onset Alzheimer’s disease, varies substantially in frequency between populations, and its explanatory power in Arab populations appears more limited than in northern European cohorts. In Wadi Ara, a highly consanguineous Israeli-Arab community with an unusually high prevalence of Alzheimer-type dementia, ε4 allele frequencies were low in both cases and controls, leading the investigators to conclude explicitly that the excess disease burden could not be attributed to ε4 [63]. Second, consanguineous marriage is common across the Gulf, with rates in Saudi Arabia frequently reported above 50% and first-cousin unions predominating [64]. Consanguinity increases genome-wide homozygosity, which can unmask recessive or additive susceptibility alleles that remain effectively invisible in outbred populations; autozygosity mapping in the same consanguineous Arab community identified candidate loci on chromosomes 9 and 12, with evidence that the chromosome 9 signal arose from excess homozygosity among cases [65].

The implication is not that the life-course framework fails in this setting, but that its genetic architecture may differ. Available evidence suggests that the relative contributions of common alleles, recessive variants, and modifiable vascular exposures may differ across populations. However, this remains untested in representative GCC cohorts and should be addressed through adequately powered regional genomic and gene–environment studies. Two caveats bound the discussion above and should be stated plainly. The Wadi Ara findings derive from a single, highly consanguineous Israeli-Arab community and cannot be generalized to Arab or GCC populations as a whole; and no adequately powered study has examined gene–environment interaction between regional genetic background and midlife cardiometabolic burden in any Gulf cohort. Establishing APOE allele frequencies in representative GCC samples, quantifying the contribution of autozygosity to dementia risk, and testing whether high metabolic burden interacts with these backgrounds are concrete and achievable priorities. National population genome programs now under way across the region, including the Saudi Human Genome Program, the Qatar Genome Programme, and the Emirati Genome Program, are assembling the population-scale reference data that such work requires [66]. Until these large-scale regional biobank studies complete their analyses, findings from localized consanguineous community studies cannot be extrapolated to GCC populations as a whole, and no regionally specific genetic contribution to dementia risk should be assumed in clinical or public-health planning. The genetic dimension is therefore best treated as hypothesis-generating, a reason to build regional cohorts, rather than as an established modifier of prevention strategy.

### 7.3. Adapting Prevention to the Regional Context

Several adaptations are likely to matter. Dietary counseling should reflect regional eating patterns rather than assume a Mediterranean baseline; physical activity advice must account for climate, gender-specific barriers to outdoor exercise, and the built environment; and tobacco and waterpipe use call for culturally specific cessation support. Health-system design is equally important, including integrating brain-health messaging into primary care and diabetes services, where most midlife patients are already seen. The central message for clinicians in these settings is that global evidence supports acting now, while the details of delivery should be tailored to local risk profiles, resources, and culture.

## 8. From Risk-Factor Lists to Clinical Prevention

The practical implication is that brain-health prevention should be integrated into routine midlife care. Neurologists, primary-care physicians, cardiologists, endocrinologists, psychiatrists, sleep physicians, dietitians, nurses, and public-health teams encounter modifiable risks long before dementia is diagnosed. A patient with midlife hypertension, diabetes, obesity, smoking, poor sleep, and inactivity should not receive fragmented messages. Instead, the patient should receive a coherent prevention plan that explains how vascular and lifestyle risks affect the heart, brain, kidneys, mobility, and future independence.

Risk communication must be accurate. It is inappropriate to promise that modifying risk factors will prevent dementia in a specific individual. Age, genetics, education, socioeconomic factors, traumatic brain injury, sensory impairment, depression, air pollution, and other exposures all contribute to risk [2]. However, it is equally inappropriate to ignore modifiable risk factors merely because causality is complex. The most ethical message is that midlife risk-factor control cannot guarantee protection, but it can reduce cumulative biological injury and improve multiple health outcomes.

Prevention should also be multidomain. Single-risk interventions are important, but dementia arises from interacting systems. The FINGER trial demonstrated that a multidomain intervention combining diet, exercise, cognitive training, social activity, and vascular monitoring can improve cognitive performance in at-risk older adults [25]. Although FINGER enrolled older adults rather than a purely midlife population, it supports the principle that combined behavioral and vascular strategies are feasible and clinically meaningful. More recently, the US POINTER trial extended this evidence to a large and diverse cohort, showing that a structured multidomain lifestyle intervention improved global cognition over two years, with broadly consistent findings across prespecified subgroups, including APOE ε4 status [26]. The next step is to adapt these models earlier in the life course and across diverse healthcare systems.

## 9. Controversies and Evolving Evidence

Several active debates qualify the prevention narrative and warrant explicit acknowledgment (Table 3). The first concerns lipids. Although high midlife LDL cholesterol is now listed among modifiable dementia risk factors [2], the therapeutic evidence is more equivocal: a 2025 meta-analysis of 20 randomized trials found that lipid-lowering therapy did not significantly reduce the incidence of dementia or cognitive impairment, even though it confirmed the cognitive safety of statins [17]. The most defensible reading is that lipid management is firmly justified for vascular protection and remains biologically relevant to brain health, but should not be presented to patients as a proven dementia prevention treatment.

A second debate concerns the advent of disease-modifying therapy. Anti-amyloid monoclonal antibodies lecanemab and donanemab have shown that removing amyloid can modestly slow clinical decline in early symptomatic Alzheimer’s disease [67,68]. These findings are important proofs of concept, but the effect sizes are modest. Treatment carries the risk of amyloid-related imaging abnormalities, and the requirements for biomarker confirmation, repeated infusions, specialist monitoring, and high cost place these therapies out of reach for most patients worldwide, particularly in low- and middle-income settings. Rather than diminishing the case for prevention, the disease-modifying era strengthens it: treatments that act late and only partially make earlier risk reduction more valuable, not less.

A third area of genuine uncertainty concerns mechanisms. The glymphatic hypothesis offers an appealing link between sleep and the clearance of neurotoxic proteins, but the foundational work was conducted largely in rodents [28], and the extent to which sleep-dependent clearance operates and matters in the human brain remains to be established. The same caution applies to the precise causal weight of any single exposure. These uncertainties do not undermine the life-course model, which rests on convergent epidemiological and mechanistic evidence, but they argue for humility about individual pathways and for continued investment in human, longitudinal, and biomarker-based research.

## 10. Limitations and Future Directions

Several limitations of the evidence base should temper interpretation. Much of the evidence linking midlife risk factors to late-life dementia comes from observational studies. These studies are vulnerable to residual confounding, measurement variability, survival bias, competing mortality, and reverse causation. For example, late-life weight loss or blood-pressure decline may reflect preclinical disease rather than protection. Definitions of midlife, dementia subtype, cognitive decline, and exposure intensity also vary across studies.

Intervention evidence remains challenging because dementia develops over decades. Trials may be too short, start too late, test single factors in isolation, or include participants with already advanced pathology. A negative or modest trial result should not be taken as proof that midlife risk modification is irrelevant. Instead, it underscores the need for longer, earlier, and more integrated prevention studies that combine vascular, metabolic, behavioral, cognitive, and social interventions.

Future research should prioritize diverse populations, including those in regions undergoing rapid demographic, nutritional, and lifestyle transitions. Many influential cohorts are based in high-income settings and may not fully capture variation in education, vascular-risk prevalence, healthcare access, air pollution, diet, sleep patterns, and family structure. Studies that integrate longitudinal clinical data, imaging, biomarkers, genomics, and implementation science will be especially useful for identifying who benefits most, when interventions should begin, and how prevention can be delivered at scale.

## 11. Conclusions

Late-life cognitive decline and dementia are shaped by exposures that often emerge or become entrenched in midlife. Hypertension, diabetes, obesity, dyslipidemia, smoking, physical inactivity, unhealthy dietary patterns, sleep disturbance, low cognitive engagement, and social disconnection interact through vascular, metabolic, inflammatory, neurodegenerative, and reserve-related pathways.

A midlife prevention framework is clinically practical and biologically plausible, but its components are not all supported to the same degree, and conclusions should be graded accordingly. Blood-pressure control carries the strongest evidence, resting on consistent cohorts together with randomized data showing benefit for mild cognitive impairment; multidomain lifestyle programs combining diet, exercise, cognitive training, social activity, and vascular monitoring have demonstrated cognitive benefit in randomized trials, although as packages rather than as separable ingredients. Recommendations concerning adiposity, diet, sleep, smoking, and social engagement rest principally on observational evidence and, for lipids, on observational and genetic evidence rather than on trials with cognitive endpoints; these remain biologically plausible and clinically reasonable, and each is independently justified by its established non-cognitive benefits, but they should be communicated as prudent rather than proven for dementia prevention. This framework does not imply that dementia is fully preventable or that responsibility rests solely with individuals. Rather, it recognizes that cumulative risk can be reduced through earlier vascular control, healthier behaviors, social and cognitive engagement, and supportive health systems. Dementia prevention should be treated as a core component of adult medicine and public health, beginning decades before the typical age of diagnosis. Nowhere is this more consequential than in the Gulf, where a high midlife burden of vascular and metabolic risk coincides with one of the steepest projected increases in dementia among world regions. Acting on these modifiable exposures now, decades before the coming wave of cases, may be among the most effective investments the Gulf states can make in their future brain health [4,57].

## Figures and Tables

**Table 1:** Major modifiable midlife determinants relevant to late-life cognitive decline and dementia.

Risk Domain	Representative Evidence	Evidence Class	Plausible Pathways	Clinical Implication
Hypertension	Associated with higher dementia risk; recent data show domain-specific effects [5,6,7,8].	Cohort evidence plus a randomized trial reporting cognitive endpoints (SPRINT MIND) [9].	Cerebral small-vessel disease and white-matter injury.	Treat blood pressure early and consistently; frame control as both cardiovascular and brain protection.
Diabetes/insulin resistance	Diabetes, glycemic variability, and insulin resistance each add risk [5,10,11,12,13].	Cohort and mechanistic evidence; no dementia-endpoint trial of glucose lowering.	Microvascular injury and brain insulin resistance.	Use integrated cardiometabolic care rather than glucose-only counseling.
Obesity/adiposity	Higher risk, driven especially by central adiposity and weight variability [6,14,15,16].	Cohort and neuroimaging evidence; observational only.	Systemic inflammation and insulin resistance.	Address weight, waist circumference, diet, activity, and sleep as a combined risk cluster.
Dyslipidemia/high LDL-C	Listed as a modifiable risk [2]; trial evidence for lipid-lowering on cognition is equivocal [17].	Cohort evidence plus Mendelian randomization [18]; late-life lipid-lowering trials neutral [17].	Atherosclerosis and impaired cerebral perfusion.	Manage lipids according to cardiovascular-risk standards with brain-health framing.
Smoking	Associated with dementia; cessation lowers risk toward never-smoker levels [5,19,20,21].	Cohort evidence only; no dementia-endpoint trial.	Endothelial injury, hypoxia, and oxidative stress.	Make cessation a repeated, supported, system-level intervention.
Physical inactivity	Associated with higher dementia risk; inverse associations between physical activity and dementia are stronger in studies with longer follow-up [22,23,24].	Cohort evidence plus multidomain intervention trials [25,26].	Reduced vascular fitness, impaired metabolic health, and diminished neurotrophic signaling.	Prescribe sustainable activity as a core component of brain-health prevention.
Sleep disturbance	Short sleep and sleep disorders are associated with higher dementia incidence [27,28,29,30].	Cohort and meta-analytic evidence; no dementia-endpoint trial.	Impaired clearance, vascular stress, and intermittent hypoxia.	Screen for insomnia and sleep-disordered breathing when clinically indicated.
Low cognitive or social engagement	Low engagement and loneliness are independently associated with dementia risk [31,32,33].	Cohort evidence plus multidomain trial components [25,26].	Lower cognitive reserve and chronic stress biology.	Promote cognitive activity and social participation as realistic preventive supports.

**Table 2:** Selected evidence informing a midlife brain-health prevention framework.

Study/Source	Design or Source Type	Exposure/Intervention	Main Relevance to This Review
Lancet Commission 2024 [2]	Expert commission and evidence synthesis	Fourteen modifiable dementia risk factors across the life course	Supports prevention across early, mid-, and late life; estimates around 45% of cases may be preventable or delayable.
Whitmer et al. [5]	Large retrospective cohort	Midlife hypertension, diabetes, smoking, and high cholesterol	Demonstrates cumulative cardiovascular risk-factor burden in midlife and subsequent dementia risk.
Kivipelto et al. [6]	Population-based cohort	Midlife obesity and vascular risk factors	Supports the importance of clustered midlife cardiometabolic exposures.
Gottesman et al. [36]	Community cohort with neuroimaging	Midlife vascular risk factors and late-life amyloid deposition	Links vascular risk with later brain biomarker and imaging outcomes.
SPRINT MIND [9]	Randomized clinical trial	Intensive versus standard systolic blood-pressure treatment	Shows cognitive signals for blood-pressure intervention, especially mild cognitive impairment outcomes.
FINGER trial [25]	Randomized multidomain intervention	Diet, exercise, cognitive training, social activity, vascular monitoring	Supports feasibility and cognitive benefit of multidomain prevention in at-risk older adults.

**Table 3:** Active debates and evolving evidence in midlife dementia prevention.

Debate/Open Question	Current Evidence	Practical Implication
Lipid-lowering and cognition	Midlife LDL-C is a listed risk factor [2], but a 2025 meta-analysis of 20 trials found lipid-lowering therapy did not significantly reduce dementia or cognitive impairment, while confirming statin cognitive safety [17].	Manage lipids for vascular protection; do not present lipid-lowering as a proven dementia prevention therapy.
Anti-amyloid disease-modifying therapy	Lecanemab and donanemab modestly slow decline in early Alzheimer’s disease [67,68], but effect sizes are small, ARIA risk and cost are substantial, and access is limited worldwide.	Late, partial treatment strengthens rather than replaces the case for earlier midlife risk reduction.
Glymphatic clearance and sleep	The glymphatic hypothesis links sleep to clearance of neurotoxic proteins, but foundational work was largely in rodents [28]; human relevance remains to be established.	Treat sleep disorders for established benefits; avoid overstating a single unproven mechanism.
Generalizability of the Lancet risk model	The Commission’s own authors acknowledge their population-attributable-fraction estimates are assumption-dependent and provisional [69]; preliminary conference-abstract findings from a single-cohort replication, not yet confirmed in a full report, further suggest that only a subset of factors remained significant when modeled jointly [70].	Interpret population risk estimates as probabilistic; replicate prevention frameworks in diverse and regional cohorts.

## Data Availability

No new data were created or analyzed in this study. Data sharing is not applicable to this article.

## References

[1] World Health Organization (2025). Dementia. Fact Sheet. https://www.who.int/news-room/fact-sheets/detail/dementia.

[2] Livingston G., Huntley J., Liu K.Y., Costafreda S.G., Selbaek G., Alladi S., Ames D., Banerjee S., Burns A., Brayne C. (2024). Dementia prevention, intervention, and care: 2024 report of the Lancet standing Commission. Lancet.

[3] Jack C.R., Bennett D.A., Blennow K., Carrillo M.C., Dunn B., Haeberlein S.B., Holtzman D.M., Jagust W., Jessen F., Karlawish J. (2018). NIA-AA Research Framework: Toward a biological definition of Alzheimer’s disease. Alzheimer’s Dement..

[4] Nichols E., Steinmetz J.D., Vollset S.E., Fukutaki K., Chalek J., Abd-Allah F., Abdoli A., Abualhasan A., Abu-Gharbieh E., Akram T.T. (2022). Estimation of the global prevalence of dementia in 2019 and forecasted prevalence in 2050: An analysis for the Global Burden of Disease Study 2019. Lancet Public Health.

[5] Whitmer R.A., Sidney S., Selby J., Johnston S.C., Yaffe K. (2005). Midlife cardiovascular risk factors and risk of dementia in late life. Neurology.

[6] Kivipelto M., Ngandu T., Fratiglioni L., Viitanen M., Kareholt I., Winblad B., Helkala E.-L., Tuomilehto J., Soininen H., Nissinen A. (2005). Obesity and vascular risk factors at midlife and the risk of dementia and Alzheimer disease. Arch. Neurol..

[7] Floud S., Hermon C., Whiteley W., Fitzpatrick K.E., Reeves G.K. (2025). Hypertension in pregnancy and in midlife and the risk of dementia: Prospective study of 1.3 million UK women. Alzheimer’s Dement..

[8] Joyce O.C., McHugh C., Mockler D., Wilson F., Kelly A.M. (2024). Midlife hypertension is a risk factor for some, but not all, domains of cognitive decline in later life: A systematic review and meta-analysis. J. Hypertens..

[9] Williamson J.D., Pajewski N.M., Auchus A.P., Bryan R.N., Chelune G., Cheung A.K., Cleveland M.L., Coker L.H., Crowe M.G., SPRINT MIND Investigators for the SPRINT Research Group (2019). Effect of intensive vs standard blood pressure control on probable dementia: A randomized clinical trial. JAMA.

[10] Biessels G.J., Staekenborg S., Brunner E., Brayne C., Scheltens P. (2006). Risk of dementia in diabetes mellitus: A systematic review. Lancet Neurol..

[11] Tabesh M., Sacre J.W., Mehta K., Chen L., Sajjadi S.F., Magliano D.J., Shaw J.E. (2025). Associations of glycaemia-related risk factors with dementia and cognitive decline in individuals with type 2 diabetes: A systematic review and meta-analysis. Diabet. Med..

[12] Biswas R., Capuano A.W., Mehta R.I., Barnes L.L., Bennett D.A., Arvanitakis Z. (2025). Review of associations of diabetes and insulin resistance with brain health in three harmonised cohort studies of ageing and dementia. Diabetes Metab. Res. Rev..

[13] Yu J.H., Kim R.E.Y., Park S.Y., Lee D.Y., Cho H.J., Kim N.H., Yoo H.J., Seo J.A., Kim S.G., Choi K.M. (2023). Association of long-term hyperglycaemia and insulin resistance with brain atrophy and cognitive decline: A longitudinal cohort study. Diabetes Obes. Metab..

[14] Anstey K.J., Cherbuin N., Budge M., Young J. (2011). Body mass index in midlife and late-life as a risk factor for dementia: A meta-analysis of prospective studies. Obes. Rev..

[15] Wei J., Zhu X., Liu J., Gao Y., Liu X., Wang K., Zheng X. (2025). Estimating global prevalence of mild cognitive impairment and dementia in elderly with overweight, obesity, and central obesity: A systematic review and meta-analysis. Obes. Rev..

[16] HasanRashedi M., Ghaemi S., Salabat D., Bafkar N., Bahrami Rad A., Vasheghani Farahani A., Gohari Dezfuli Z., Memari A. (2026). The association between body weight variability and dementia and cognitive decline: A systematic review and meta-analysis of cohort studies with the GRADE assessment. Obes. Rev..

[17] Reddin C., Stankard A., Chan K.Y., Krewer F., Judge C., Canavan M., Davis D.H.J., O’Donnell M. (2025). Association of lipid-lowering therapy with dementia and cognitive outcomes: A systematic review and meta-analysis. Age Ageing.

[18] Nordestgaard L.T., Hanson A., Sanderson E., Anderson E., Walker V., Tybjærg-Hansen A., Davey Smith G., Nordestgaard B.G. (2025). Cholesterol-lowering drug targets reduce risk of dementia: Mendelian randomization and meta-analyses of 1 million individuals. Alzheimer’s Dement..

[19] Peters R., Poulter R., Warner J., Beckett N., Burch L., Bulpitt C. (2008). Smoking, dementia and cognitive decline in the elderly, a systematic review. BMC Geriatr..

[20] Chen H., Wang J., Lai S., Peng G., Zong G., Yuan C., Luo B. (2026). Smoking cessation, weight change, and risk of dementia: A prospective cohort study. Neurology.

[21] Bloomberg M., Brown J., Di Gessa G., Bu F., Steptoe A. (2025). Cognitive decline before and after mid-to-late-life smoking cessation: A longitudinal analysis of prospective cohort studies from 12 countries. Lancet Healthy Longev..

[22] Rovio S., Kareholt I., Helkala E.-L., Viitanen M., Winblad B., Tuomilehto J., Soininen H., Nissinen A., Kivipelto M. (2005). Leisure-time physical activity at midlife and the risk of dementia and Alzheimer’s disease. Lancet Neurol..

[23] Hamer M., Chida Y. (2009). Physical activity and risk of neurodegenerative disease: A systematic review of prospective evidence. Psychol. Med..

[24] Iso-Markku P., Kujala U.M., Knittle K., Polet J., Vuoksimaa E., Waller K. (2022). Physical activity as a protective factor for dementia and Alzheimer’s disease: Systematic review, meta-analysis and quality assessment of cohort and case-control studies. Br. J. Sports Med..

[25] Ngandu T., Lehtisalo J., Solomon A., Levalahti E., Ahtiluoto S., Antikainen R., Backman L., Hanninen T., Jula A., Laatikainen T. (2015). A 2 year multidomain intervention of diet, exercise, cognitive training, and vascular risk monitoring versus control to prevent cognitive decline in at-risk elderly people (FINGER): A randomised controlled trial. Lancet.

[26] Baker L.D., Espeland M.A., Whitmer R.A., Snyder H.M., Leng X., Lovato L., Papp K.V., Yu M., Kivipelto M., Alexander A.S. (2025). Structured vs self-guided multidomain lifestyle interventions for global cognitive function: The US POINTER randomized clinical trial. JAMA.

[27] Sabia S., Fayosse A., Dumurgier J., van Hees V.T., Paquet C., Sommerlad A., Kivimaki M., Dugravot A., Singh-Manoux A. (2021). Association of sleep duration in middle and old age with incidence of dementia. Nat. Commun..

[28] Xie L., Kang H., Xu Q., Chen M.J., Liao Y., Thiyagarajan M., O’Donnell J., Christensen D.J., Nicholson C., Iliff J.J. (2013). Sleep drives metabolite clearance from the adult brain. Science.

[29] Ungvari Z., Fekete M., Lehoczki A., Munkacsy G., Fekete J.T., Zabo V., Purebl G., Varga P., Ungvari A., Gyorffy B. (2025). Sleep disorders increase the risk of dementia, Alzheimer’s disease, and cognitive decline: A meta-analysis. Geroscience.

[30] Miyata J., Muraki I., Iso H., Yamagishi K., Yasuda N., Sawada N., Inoue M., Tsugane S. (2024). Sleep duration, its change, and risk of dementia among Japanese: The Japan Public Health Center-based prospective study. Prev. Med..

[31] Stern Y. (2012). Cognitive reserve in ageing and Alzheimer’s disease. Lancet Neurol..

[32] Kuiper J.S., Zuidersma M., Oude Voshaar R.C., Zuidema S.U., van den Heuvel E.R., Stolk R.P., Smidt N. (2015). Social relationships and risk of dementia: A systematic review and meta-analysis of longitudinal cohort studies. Ageing Res. Rev..

[33] Luchetti M., Aschwanden D., Sesker A.A., Zhu X., O’Suilleabhain P.S., Stephan Y., Terracciano A., Sutin A.R. (2024). A meta-analysis of loneliness and risk of dementia using longitudinal data from >600,000 individuals. Nat. Ment. Health.

[34] Norton S., Matthews F.E., Barnes D.E., Yaffe K., Brayne C. (2014). Potential for primary prevention of Alzheimer’s disease: An analysis of population-based data. Lancet Neurol..

[35] Deckers K., van Boxtel M.P.J., Schiepers O.J.G., de Vugt M., Sanchez J.L., Anstey K.J., Brayne C., Dartigues J.-F., Engedal K., Kivipelto M. (2015). Target risk factors for dementia prevention: A systematic review and Delphi consensus study on the evidence from observational studies. Int. J. Geriatr. Psychiatry.

[36] Gottesman R.F., Schneider A.L.C., Zhou Y., Coresh J., Green E., Gupta N., Knopman D.S., Mintz A., Rahmim A., Sharrett A.R. (2017). Association between midlife vascular risk factors and estimated brain amyloid deposition. JAMA.

[37] Debette S., Seshadri S., Beiser A., Au R., Himali J.J., Palumbo C., Wolf P.A., DeCarli C. (2011). Midlife vascular risk factor exposure accelerates structural brain aging and cognitive decline. Neurology.

[38] Aderinto N., Olatunji G., Abdulbasit M., Ashinze P., Faturoti O., Ajagbe A., Ukoaka B., Aboderin G. (2023). The impact of diabetes in cognitive impairment: A review of current evidence and prospects for future investigations. Medicine.

[39] Kim A.B., Arvanitakis Z. (2023). Insulin resistance, cognition, and Alzheimer’s disease. Obesity.

[40] Raji C.A., Meysami S., Hashemi S., Garg S., Akbari N., Gouda A., Chodakiewitz Y.G., Nguyen T.D., Niotis K., Merrill D.A. (2024). Visceral and subcutaneous abdominal fat predict brain volume loss at midlife in 10,001 individuals. Aging Dis..

[41] Xu S., Wen S., Yang Y., He J., Yang H., Qu Y., Zeng Y., Zhu J., Fang F., Song H. (2024). Association between body composition patterns, cardiovascular disease, and risk of neurodegenerative disease in the UK Biobank. Neurology.

[42] Fang S., Guan L., Jian H., Dai X.J., Gong L. (2025). Body weight and BMI variability linked to dementia risk: A meta-analysis. Biomol. Biomed..

[43] Stephan B.C.M., Cochrane L., Kafadar A.H., Brain J., Burton E., Myers B., Brayne C., Naheed A., Anstey K.J., Ashor A.W. (2024). Population attributable fractions of modifiable risk factors for dementia: A systematic review and meta-analysis. Lancet Healthy Longev..

[44] Morris M.C., Tangney C.C., Wang Y., Sacks F.M., Barnes L.L., Bennett D.A., Aggarwal N.T. (2015). MIND diet associated with reduced incidence of Alzheimer’s disease. Alzheimer’s Dement..

[45] Scarmeas N., Stern Y., Tang M.-X., Mayeux R., Luchsinger J.A. (2006). Mediterranean diet and risk for Alzheimer’s disease. Ann. Neurol..

[46] Huang L., Tao Y., Chen H., Chen X., Shen J., Zhao C., Xu X., He M., Zhu D., Zhang R. (2023). Mediterranean-Dietary Approaches to Stop Hypertension Intervention for Neurodegenerative Delay (MIND) diet and cognitive function and its decline: A prospective study and meta-analysis of cohort studies. Am. J. Clin. Nutr..

[47] Sawyer R.P., Blair J., Shatz R., Manly J.J., Judd S.E. (2024). Association of adherence to a MIND-style diet with the risk of cognitive impairment and decline in the REGARDS cohort. Neurology.

[48] Agarwal P., Leurgans S.E., Agrawal S., Aggarwal N.T., Cherian L.J., James B.D., Dhana K., Barnes L.L., Bennett D.A., Schneider J.A. (2024). Association of MIND diet with cognitive decline among Black and White older adults. Alzheimer’s Dement..

[49] Charisis S., Yannakoulia M., Scarmeas N. (2024). Diets to promote healthy brain ageing. Nat. Rev. Neurol..

[50] Zhang J., Ou J., Lu X., Wang T., Dang W., Ding L., Liu Y., Yan B., Yu H. (2025). Sleep disorders and the risk of cognitive decline or dementia: An updated systematic review and meta-analysis of longitudinal studies. J. Neurol..

[51] Mahalingam G., Samtani S., Lam B.C.P., Lipnicki D.M., Lima-Costa M.F., Blay S.L., Castro-Costa E., Shifu X., Guerchet M., Preux P.-M. (2023). Social connections and risk of incident mild cognitive impairment, dementia, and mortality in 13 longitudinal cohort studies of ageing. Alzheimer’s Dement..

[52] Huang A.R., Roth D.L., Cidav T., Chung S.E., Amjad H., Thorpe R.J., Boyd C.M., Cudjoe T.K.M. (2023). Social isolation and 9-year dementia risk in community-dwelling Medicare beneficiaries in the United States. J. Am. Geriatr. Soc..

[53] El-Metwally A., Toivola P., Al-Rashidi M., Nooruddin S., Jawed M., AlKanhal R., Abdul Razzak H., Albawardi N. (2019). Epidemiology of Alzheimer’s disease and dementia in Arab countries: A systematic review. Behav. Neurol..

[54] Hajjar S.H., Alsaad S.M., AlFouzan M.A., Rohaiem S.N., Alamri S.H., Altamimi T.N., Alodhayani A.A., Hassanin H.I., AlHarkan K.S., Albalawi A.A. (2025). Dementia prevalence within the Kingdom of Saudi Arabia National Guard Health System (2015–2023): An exploratory epidemiological study. J. Alzheimer’s Dis..

[55] Qassem T., Itani L., Nasr W. (2023). Prevalence and economic burden of dementia in the Arab world. BJPsych Open.

[56] Boettiger D.C., Lin T.K., Almansour M., Hamza M.M., Alsukait R., Herbst C.H., Altheyab N., Afghani A., Kattan F. (2023). Projected impact of population aging on non-communicable disease burden and costs in the Kingdom of Saudi Arabia, 2020–2030. BMC Health Serv. Res..

[57] Qadi N., Alkhodair Y., Alshammari W., Aldakheel A. (2025). Epidemiological and economic burden of dementia in the Middle East and North African region. J. Alzheimer’s Dis..

[58] Al-Thani M.H.J.T., El-Saharty S., Jamal Z., Mishra A., Kokku S.B., Nawaz R.N., Skaroni I., Abou-Samra A.B. (2025). Qatar’s progress in curbing diabetes: A comprehensive and proactive approach. Health Syst. Reform.

[59] Mezhal F., Oulhaj A., Abdulle A., AlJunaibi A., Alnaeemi A., Ahmad A., Leinberger-Jabari A., Al Dhaheri A.S., AlZaabi E., Al-Maskari F. (2023). High prevalence of cardiometabolic risk factors amongst young adults in the United Arab Emirates: The UAE Healthy Future Study. BMC Cardiovasc. Disord..

[60] Chaabane S., Chaabna K., Abraham A., Mamtani R., Cheema S. (2020). Physical activity and sedentary behaviour in the Middle East and North Africa: An overview of systematic reviews and meta-analysis. Sci. Rep..

[61] Al-Zalabani A.H. (2024). Secondhand smoke exposure among adolescents in the Gulf Cooperation Council countries: Analysis of Global Youth Tobacco Surveys. Sci. Rep..

[62] Alkhunizan M., Alkhenizan A., Basudan L. (2018). Prevalence of mild cognitive impairment and dementia in Saudi Arabia: A community-based study. Dement. Geriatr. Cogn. Dis. Extra.

[63] Bowirrat A., Friedland R.P., Chapman J., Korczyn A.D. (2000). The very high prevalence of AD in an Arab population is not explained by APOE ε4 allele frequency. Neurology.

[64] Khayat A.M., Alshareef B.G., Alharbi S.F., AlZahrani M.M., Alshangity B.A., Tashkandi N.F. (2024). Consanguineous marriage and its association with genetic disorders in Saudi Arabia: A review. Cureus.

[65] Farrer L.A., Bowirrat A., Friedland R.P., Waraska K., Korczyn A.D., Baldwin C.T. (2003). Identification of multiple loci for Alzheimer disease in a consanguineous Israeli-Arab community. Hum. Mol. Genet..

[66] Ateia H., Ogrodzki P., Wilson H.V., Ganesan S., Halwani R., Koshy A., Zaher W.A. (2023). Population genome programs across the Middle East and North Africa: Successes, challenges, and future directions. Biomed. Hub.

[67] van Dyck C.H., Swanson C.J., Aisen P., Bateman R.J., Chen C., Gee M., Kanekiyo M., Li D., Reyderman L., Cohen S. (2023). Lecanemab in early Alzheimer’s disease. N. Engl. J. Med..

[68] Sims J.R., Zimmer J.A., Evans C.D., Lu M., Ardayfio P., Sparks J., Wessels A.M., Shcherbinin S., Wang H., Monkul Nery E.S. (2023). Donanemab in early symptomatic Alzheimer disease: The TRAILBLAZER-ALZ 2 randomized clinical trial. JAMA.

[69] Livingston G., Costafreda S.G., Kivimäki M., Mukadam N., Schneider L.S., Selbæk G., Shirai K., Sommerlad A. (2025). Reflections on The Lancet’s Commission on dementia prevention, intervention, and care—Authors’ reply. Lancet.

[70] Williams V.J., Trane R., Sicinski K., Roan C., Herd P., Engelman M., Asthana S. (2025). Life course modifiable risk factors of dementia: Replicating the 2024 Lancet Commission model in a single longitudinal cohort. Alzheimer’s Dement..

